# Structure Elucidation and Toxicity Analysis of the Byproducts Formed after Biodegradation of Aflatoxins B1 and B2 Using Extracts of *Mentha arvensis*

**DOI:** 10.3390/toxins14010024

**Published:** 2022-01-01

**Authors:** Tehmina Anjum, Wajiha Iram, Mazhar Iqbal, Mateen Abbas, Waheed Akram, Guihua Li

**Affiliations:** 1 Guangdong Key Laboratory for New Technology Research of Vegetables, Vegetable Research Institute, Guangdong Academy of Agricultural Sciences, Guangzhou 510640, China; tehminaanjum@yahoo.com (T.A.); meher_waheed@yahoo.com (W.A.); 2 Department of Plant Pathology, Faculty of Agricultural Sciences, University of the Punjab, Lahore 54000, Pakistan; wajiha_iram@yahoo.com; 3 Health Biotechnology Division, National Institute for Biotechnology and Genetic Engineering, Faisalabad 38000, Pakistan; hamzamgondal@yahoo.com; 4 Quality Operations Laboratory, Department of Toxicology, University of Veterinary and Animal Sciences, Lahore 54000, Pakistan; mateen.abbas@uvas.edu.pk; 5 BECS Analytics and Innovation Research Boulevard, Lahore 54000, Pakistan

**Keywords:** mycotoxins, maize, mass spectrometry, HPLC, *Aspergillus flavus*

## Abstract

The aqueous extracts of leaves and shoots of *Mentha arvensis* were checked for their potential to biodegrade aflatoxin B1 and B2 (AFB1; 100 µg/L and AFB2; 50 µg/L) through in vitro assays. Overall, the results showed that leaf extract degrades aflatoxins more efficiently than the shoot extract. First, the pH, temperature and incubation time were optimized for maximum degradation by observing this activity at different temperatures between 25 and 60 °C, pH between 2 and 10 and incubation time from 3 to 72 h. In general, an increase in all these parameters significantly increased the percentage of biodegradation. In vitro trials on mature maize stock were performed under optimized conditions, i.e., pH 8, temperature 30 °C and an incubation period of 72 h. The leaf extract resulted in 75% and 80% biodegradation of AFB1 and AFB2, respectively. Whereas the shoot extract degraded both toxins up to 40–48%. The structural elucidation of degraded toxin products by LCMS/MS analysis showed seven degraded products of AFB1 and three of AFB2. MS/MS spectra showed that most of the products were formed by the loss of the methoxy group from the side chain of the benzene ring, the removal of the double bond in the terminal furan ring and the modification of the lactone group, indicating less toxicity compared to the parent compounds. The degraded products showed low toxicity against brine shrimps, confirming that *M. arvensis* leaf extract has significant potential to biodegrade aflatoxins.

## 1. Introduction

Aflatoxins comprise a family of extremely toxic mycotoxins produced by *Aspergillus flavus*, *A. parasiticus* and *A. nominus*. These are hepatotoxic mycotoxins usually produced in various agricultural commodities. Maize, however, has shown a higher contamination rate as it provides an excellent substrate for mold infection. Among the eighteen different types of aflatoxins identified so far, aflatoxins B1, B2, G1 and G2 have been reported in maize. The order of toxicity is AFB1 > AFG1 > AFB2 > AFG2, probably because of the slight difference in structures of these aflatoxins [1]. AFB1 and AFG1 contain a double bond that undergoes reduction, forming vinyl ether at the terminal furan ring; this is not the case in AFB2 and AFG2.

The problem of mycotoxin contamination in maize is more common in tropical and subtropical regions of the world because of hot and humid conditions in these areas. Tabuc, et al. [2] found 30% of the maize samples contaminated with aflatoxins B1 in a survey between 2002 and 2004 in Southeastern Romania. Similarly, Ghiasian, et al. [3], in 2011, analyzed maize samples from the Kermanshah and Mazandaran provinces of Iran and found aflatoxin contamination between 58% and 80%. Various surveys have been performed in Pakistan covering the provinces of Punjab and Khaybar Pakhtun Khuah. Scientists have reported aflatoxin levels ranging between 30 and 200 µg/kg [4,5,6,7]. In our recent survey encompassing maize storehouses of fifteen districts belonging to three agro-ecological zones of Punjab, Pakistan, 78% of collected samples were found to be contaminated with aflatoxins B1 and B2. However, aflatoxins G1, G2 and ochratoxin A were not found in any sample [8].

Various physical and chemical methods have been investigated for reducing these carcinogenic toxins to safe levels [9]. Physical methods use binding agents, such as clay, zeolites, sepiolite, kaolin, bentonites, monomorillonite and activated charcoal. The process is expensive and laborious and reduces the toxin to only 50–60%. A higher inclusion of clay can increase the binding of these compounds with minerals and antibiotics. Most of these binders are not biodegradable; thus, they can cause environmental problems. Chemical methods use caustic soda, ammonia, oxidants (such as ozone, hydrogen peroxide and sodium hypochlorite), reducing agents (such as chlorinated agents, bisulphites and formaldehyde). These chemicals are not safe, hence are not accepted by consumers [10]. Many researchers have tested the potential of microorganisms, including bacteria, yeast and fungi, to degrade these mycotoxins [11]. Microbial degradation of AFB1 using *Flavobacterium aurantiacum* (now called *Nocardia corynebacterioides*) was first reported in 1966 [12]. Though detoxification products were not identified, the residual toxicity was found to be absent when checked against ducklings [12]. After this first report, several investigations have concentrated on the biodegradation of AFB1. However, very few reports describe details regarding the degraded products and their toxicity. Many of these studies have shown the transformation of aflatoxins B1 to aflatoxicol, which is eighteen times less toxic than AFB1 but is still hazardous for other organisms [13].

Phytochemicals are of interest as a rich source of natural, ecofriendly antimicrobial components. Recently, many plant sources have been exploited for the detoxification and biodegradation of mycotoxins [14,15,16,17,18]. In this study, we investigated the potential of a common herb, i.e., *Mentha arvensis* (family: Lamiaceae), to degrade AFB1 and AFB2. *Mentha arvensis* is a well-known appetizer in Ayurveda and is traditionally used to treat digestive problems and cough [19]. A large number of different chemicals identified in this herb, including α-menthol, neomenthol, d-menthone, menthofuran, isomnethone, isomenthol, p-cymene, menthylacetate, cineol, limonine, phellandrene, aromadendrene, α-pinene, α-phellandrene, pinene, piperitone, carvomenthone, carvacrol, thujone, dipentene, cadinene, menthofuran, linalyl acetate, carvone, linalool and piperitenone oxide, are being used in pharmaceuticals, food, flavoring, cosmetics, beverages and allied industries [20,21,22]. Earlier studies have also confirmed the antifungal potential of *M. arvensis* [23,24]. Keeping this in view, the present study was planned to explore the biodegrading potential of *M. arvensis* against aflatoxins B1 and B2. The study was extended to the identification of the byproducts and evaluating their biological toxicity.

## 2. Results

### 2.1. Effect of Temperature on In Vitro Biodegradation of Aflatoxins B1 and B2 by Extracts of Mentha Arvensis

**Figure 1:**
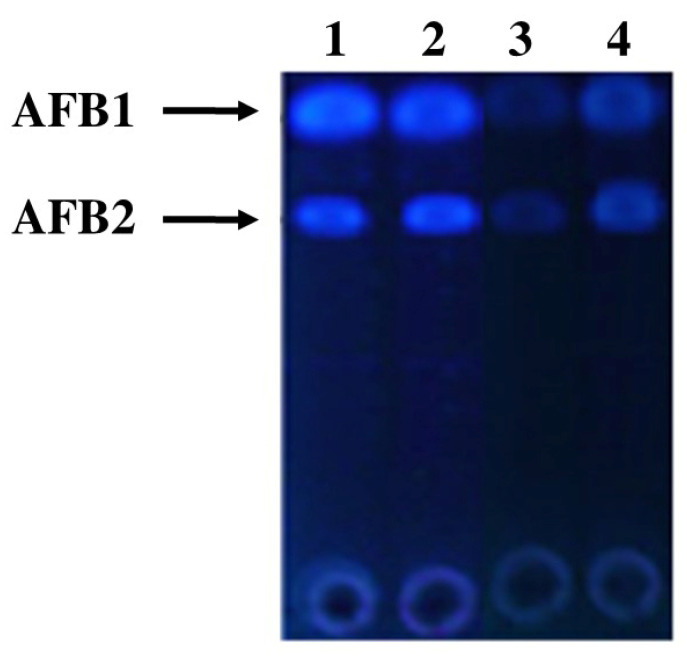
TLC analysis of control and treated toxins at 30 °C after 72 h of incubation. Where, 1 = Control (Toxin); 2 = Control (Toxin + water); 3 = Toxin + *Mentha arvensis* leaf extract; 4 = Toxin + *Mentha arvensis* shoot extract.

Time course investigation on aflatoxin degradation revealed the start of detoxification within three hours of incubation and a significant increase with an increase in incubation time. Qualitative analysis of degraded toxins by thin-layer chromatography showed a distinct decline in florescence with an increase in biodegradation percentage (Figure 1).

At the lowest tested temperature of 25 °C, the leaf extract of *M. arvensis* resulted in 44.31% and 62.16% degradation of AFB1 and AFB2, respectively, when checked after 3 h of incubation. The percentage of degradation increased with an increase in incubation time, as after 72 h, 70.9% of AFB1 and 71.85% of AFB2 were degraded at 25 °C.

Similarly, an increase in temperature significantly increased the efficacy of *M. arvensis* to biodegrade aflatoxins (Table 1). At 60 °C, the percentage of degradation in both AFB1 and AFB2 was up to 83–88%. Shoot extract was found to be less effective than the leaf extract, as after 72 h at 60 °C, it degraded AFB1 up to 50.03% and AFB2 up to 57.46%. However, further in vitro studies were carried out at 30 °C to avoid any harmful effect of high temperature on maize. In this trial, the leaf extract of *M. arvensis* at 30 °C resulted in significant degradation of AFB1 (72.12%) and AFB2 (74.01%) after an incubation of 72 h.

### 2.2. Effect of pH on In Vitro Biodegradation of Aflatoxins B1 and B2 by Extracts of M. arvensis

The results present a significant (*p* < 0.05) biodegradation in both aflatoxin B1 and B2 when incubated at a pH of 2. However, an increase in pH increased this percentage. Similarly, an increase in incubation period also enhanced the biodegradation potential of *Mentha arvensis*. The leaf extract of *M. arvensis* resulted in more degradation when compared to that of its shoot extract. After three hours of incubation, the leaf extract of *M. arvensis* caused 43.8% and 56.9% degradation of AFB1 and AFB2, respectively, at a pH of 2. With an increase in pH, this percentage increased, and at the highest tested pH of 10 a degradation of 69% was recorded in both AFB1 and AFB2 (Table 2 and Table 3). The overall results showed a maximum degradation at pH 10. However, aflatoxins are known to become sensitive and unstable at very high basic pH. Hence to avoid this in further trials, pH 8 was selected, which is much less alkaline than pH 10. The degradation percentages of both AFB1 and AFB2 at pH 8 were also found to be comparable with the results recorded at pH 10.

### 2.3. In Vitro Biodegradation of Aflatoxin B1 and B2 in Maize Samples

A similar trend of degradation was recorded in in vitro trials using mature maize stock. This study was conducted in optimized conditions, i.e., a pH of 8, temperature of 30 °C and an incubation time of 72 h. In the control, maize samples were spiked with 100 µg/L AFB1 and 50 µg/L AFB2; the toxin recovery was 97.3 and 47.6 µg/L. When the spiked maize was treated with leaf extract of *M. arvensis,* the toxin recovery reduced to 24.9 µg/L for AFB1 and 9.6 µg/L for AFB2, respectively. The potential of the shoot extract was found to be lower than the leaf extract. Its percentage biodegradation was 40% in the case of AFB1 and 46.5% for AFB2 (Table 4).

### 2.4. Mass Spectral Identification of Degraded Products of AFB1 and AFB2 Treated with M. arvensis Leaf Extracts

**Figure 2:**
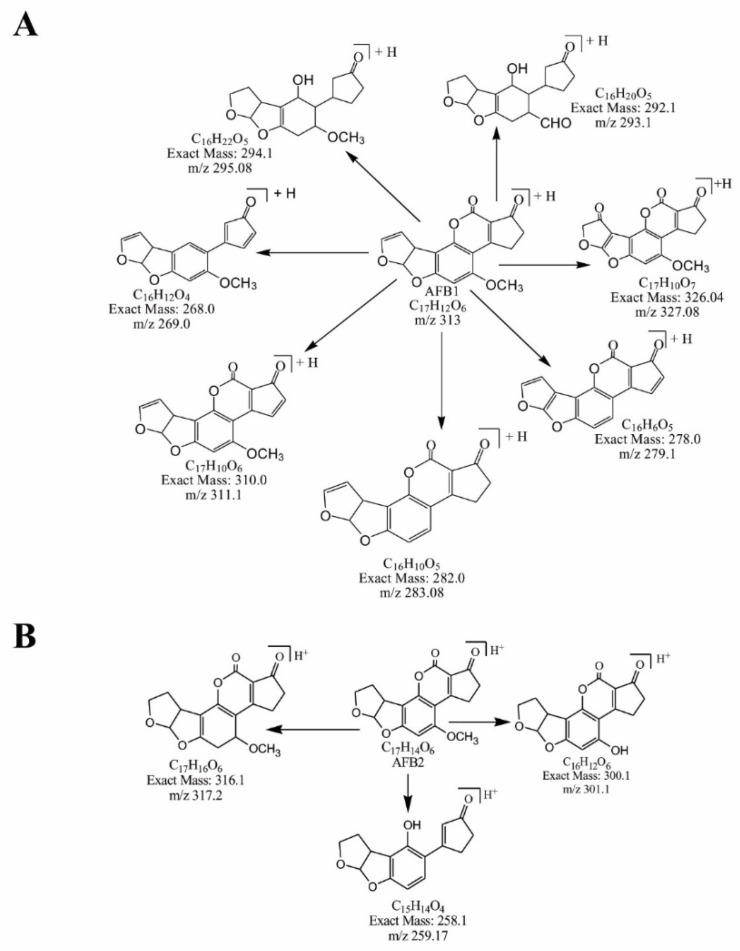
Identified degraded products of (**A**) AFB1 and (**B**) AFB2 after incubation with leaf extracts of *M. arvensis*.

The degradation products of AFB1 and AFB2 in response to the treatment with *M. arvensis* leaf extract showed structural alteration in the parent compound. The structural formulas of the identified degraded products of AFB1 and AFB2 are shown in Figure 2A,B. Leaf extract degraded aflatoxin B1 into seven new compounds and aflatoxin B2 into three products.

### 2.5. MS/MS Analysis for Confirmation of AFB1degraded Products

**Figure 3:**
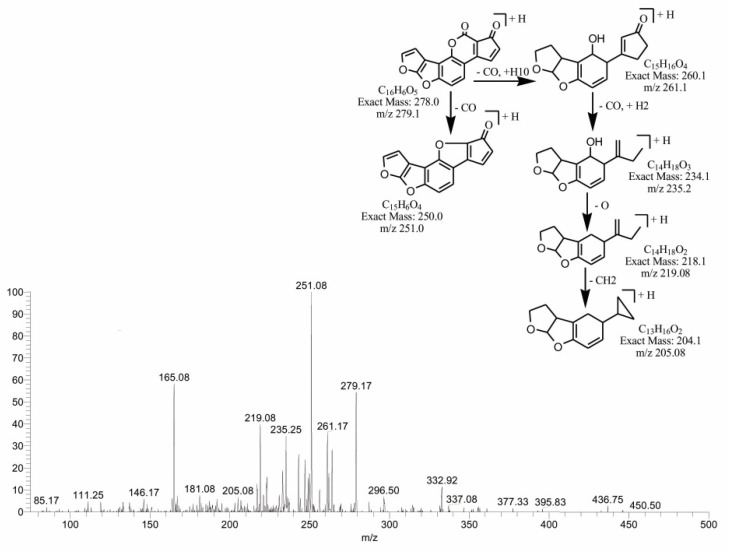
MS/MS spectra and fragmentation pathway of degradation product with 279.17 *m*/*z*.

**Figure 4:**
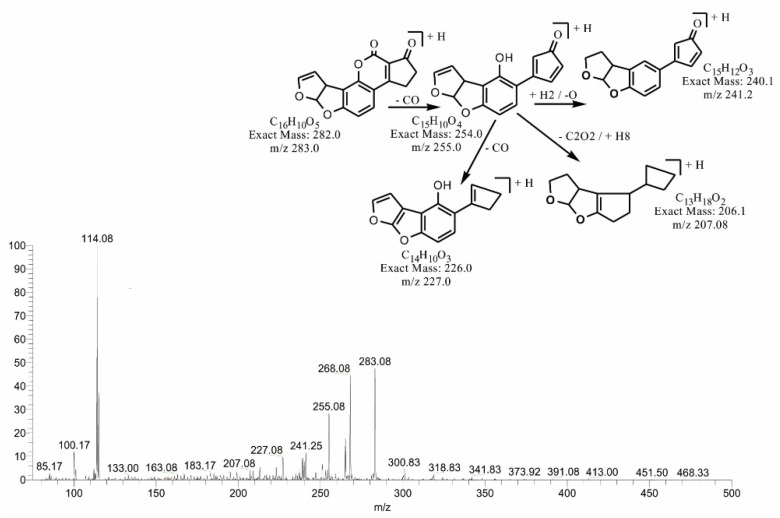
MS/MS spectra and fragmentation pathway of degraded product with 283.08 *m*/*z*.

The degraded products at *m*/*z* 279.17 and *m*/*z* 283.08 corresponded to molecular formulas C_16_H_6_O_5_ and C_16_H_10_O_5_. Both products were obtained by the loss of the methoxy group from the side chain of the benzene ring, with a difference of four hydrogen atoms. The double bond equivalence (DBE) of C_16_H_10_O_5_ was the same as that of AFB1, while C_16_H_6_O_5_ showed a DBE content greater than AFB1, i.e., 14. The loss of carbon monoxide and oxygen was the main fragmentation pathway of both products. More detail on the fragmentation of precursor ions is shown in Figure 3 and Figure 4.

**Figure 5:**
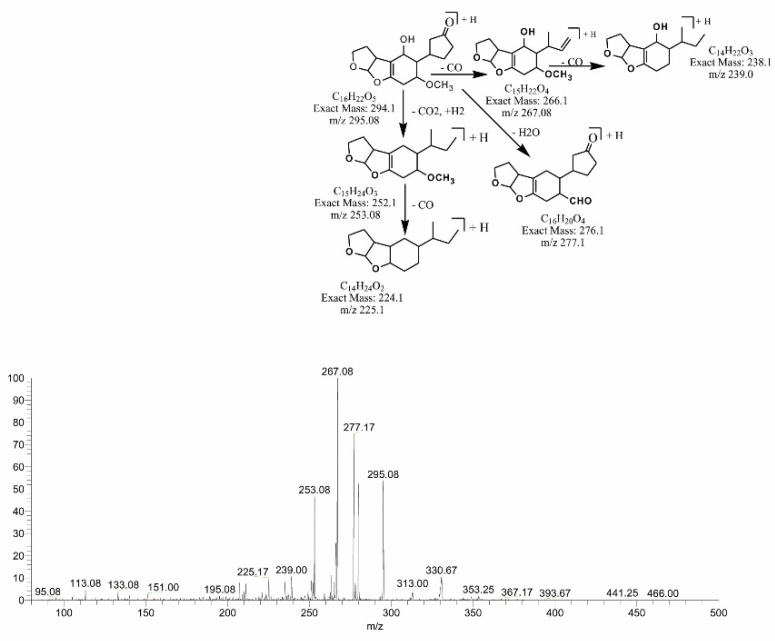
MS/MS spectra and fragmentation pathway of degraded product with 295.08 *m*/*z*.

**Figure 6:**
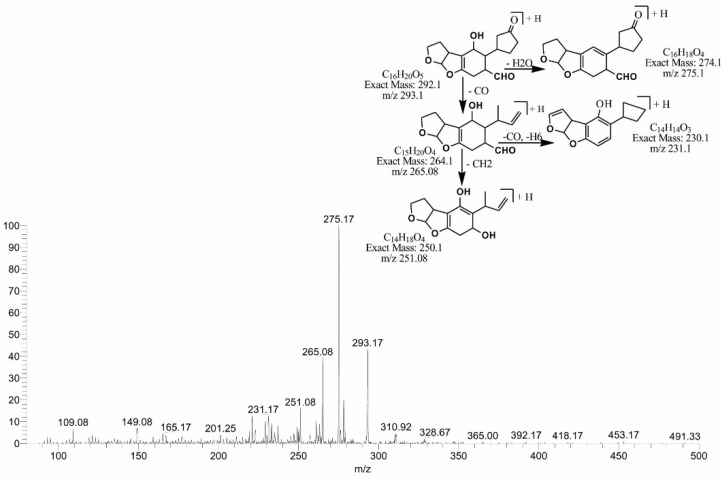
MS/MS spectra and fragmentation pathway of degraded product with 293.17 *m*/*z*.

Similarly, the degraded products obtained at 295.08 (C_16_H_22_O_5_) and 293.17 *m*/*z* (C_16_H_20_O_5_) were produced by the loss of CO from the lactone ring of the parent compound. The difference between the two products is of two hydrogen atoms with the same DBE content less than AFB1, i.e., 6. MS/MS analysis of the precursor ion C_16_H_22_O_5_ showed product ions represented by 277.17[M-H_2_O]+, 267.08[M-CO]+, 253.08[M-CO_2_]+, 239.0[M-C_2_O_2_]+ and 225.17[M-C_2_O_3_]+ (+2H). While precursor ion C_16_H_20_O_5_ yielded a series of product ions represented by 275.17[M-H_2_O]+, 265.08[M-CO]+, 251.08[M-C_2_H_2O_]+ and 231.17[M-C_2_H_6_O_2_]+ (Figure 5 and Figure 6).

**Figure 7:**
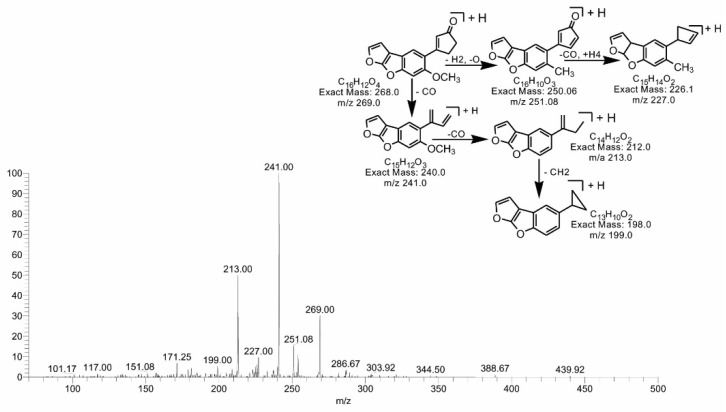
MS/MS spectra and fragmentation pathway of degraded product with 269.00 *m*/*z*.

**Figure 8:**
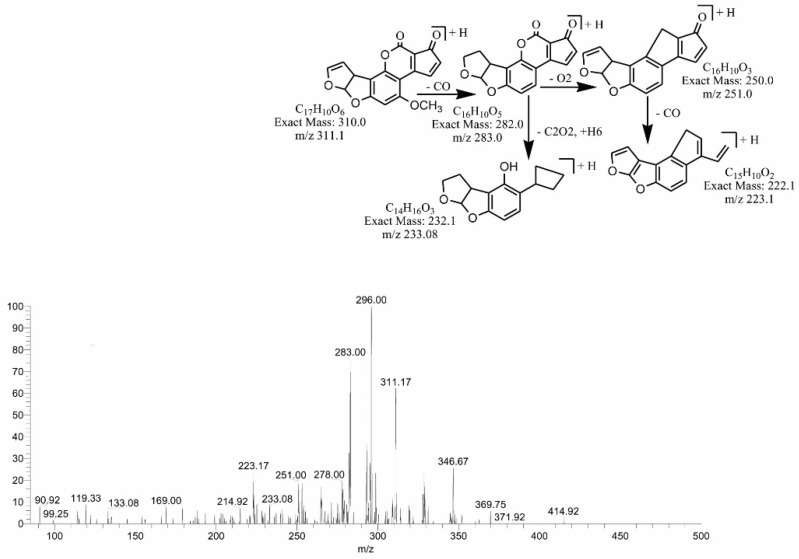
MS/MS spectra and fragmentation pathway of degraded product with 311.17 *m*/*z*.

**Figure 9:**
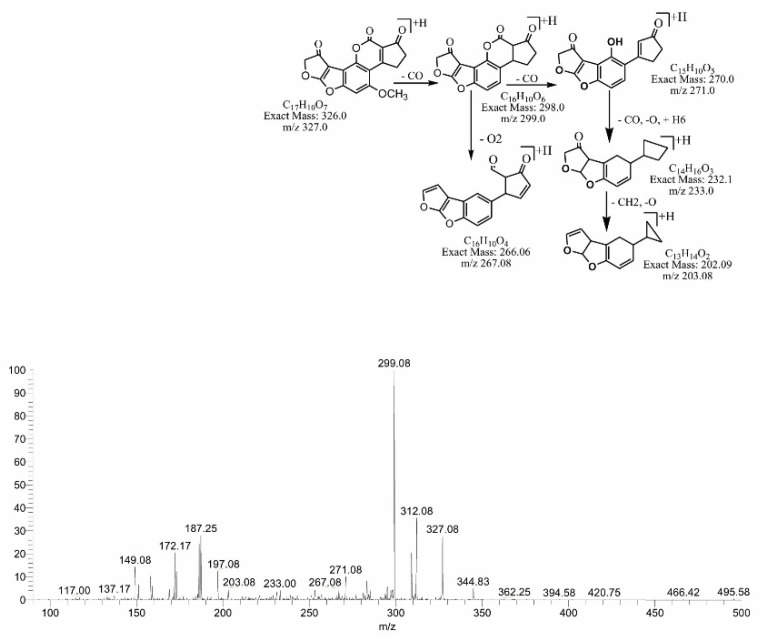
MS/MS spectra and fragmentation pathway of degraded product with 327.08 *m*/*z*.

The degradation product C_16_H_12_O_4_ (with 269.00 *m*/*z*) was formed by the loss of carbon dioxide from the lactone ring. The DBE of C_16_H_12_O_4_ was one less than AFB1. Loss of CO was the main fragmentation pathway. More details on the fragmentation pathway are shown in Figure 7. While the product obtained at 311.17 *m*/*z* (C_17_H_10_O_6_) had two more hydrogen atoms than AFB1, the DBE content of C_17_H_10_O_6_ was one more than AFB1, i.e., 13. Fragments of the precursor ion showed losses of CO, CH_2_ and O (Figure 8). The degradation product C_17_H_10_O_7_ (with *m*/*z* 327.0) was formed by the addition of an oxygen atom onto the double bond of the furan ring; the DBE content was greater than AFB1, i.e., 13. The precursor ion yielded a series of product ions, i.e., [M-CO]+, [M-CO_2_]+ and [M-C_2_O_2_]+ (Figure 9).

### 2.6. MS/MS Analysis for Confirmation of Degraded Products of AFB2

**Figure 10:**
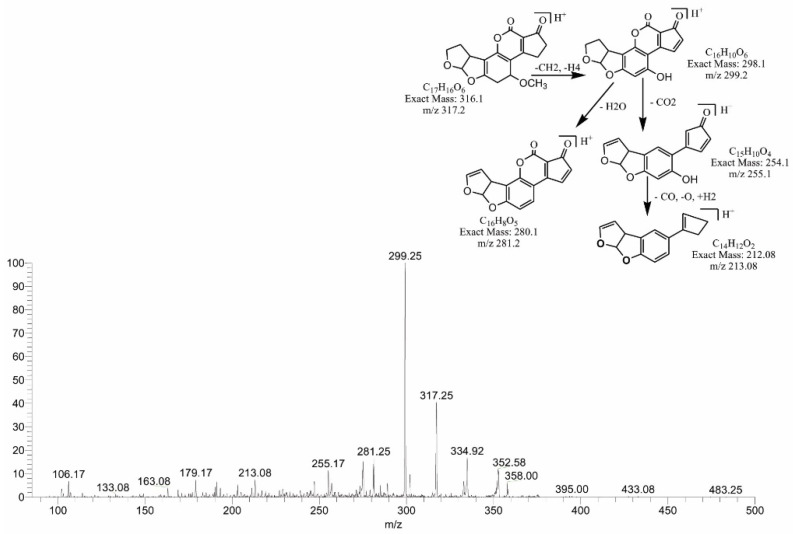
MS/MS spectra and fragmentation pathway of degraded product with 317.25 *m*/*z*.

The degraded products of AFB2 obtained at 317.25, 301.17 and 259.17 *m*/*z* corresponded to molecular formulas C_17_H_16_O_6_, C_16_H_12_O_6_ and C_15_H_14_O_4_, respectively. The product C_17_H_16_O_6_ was formed by the addition of two hydrogen atoms to the AFB2 molecule with DBE content less than that of AFB2, i.e., 10. The fragmentation pathway of C_17_H_16_O_6_ showed that precursor ions yielded a series of product ions, i.e., 299.25[M-CH_6_]+, 281.25[M-CH_8_O]+, 255.17[M-C_2_H_6_O_2_]+ and 213.08[M-C_3_H_4_O_4_]+ (Figure 10).

**Figure 11:**
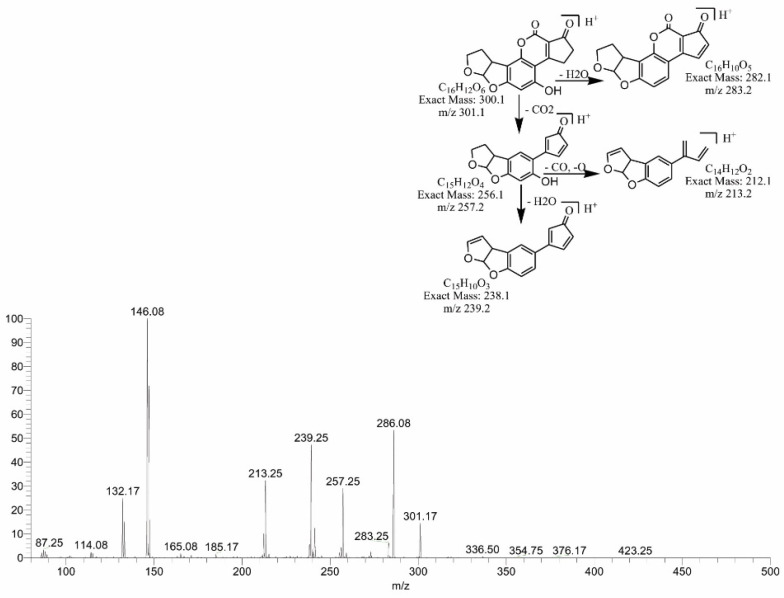
MS/MS spectra and fragmentation pathway of degraded product with 301.17 *m*/*z*.

**Figure 12:**
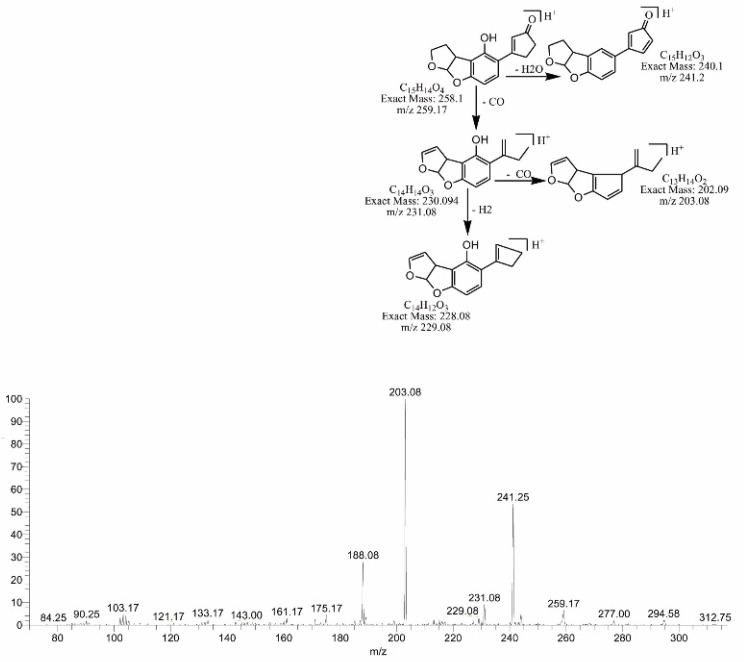
MS/MS spectra and fragmentation pathway of degraded product with 259.17 *m*/*z*.

Similarly, by the replacement of the methoxy group with the hydroxyl group on the side chain of the benzene ring, degradation product C_16_H_12_O_6_ was formed. The DBE of C_16_H_12_O_6_ was the same as that of AFB2. Fragments of 301.17 *m*/*z* showed losses of H_2_O, CO_2_ and CO (Figure 11). The degradation product C_15_H_14_O_4_ was originated by the removal of CO from the lactone ring of AFB2, with a DBE content less than AFB2, i.e., 9. The loss of CO and H_2_O was the main fragmentation pathway of C_15_H_14_O_4_ (Figure 12).

### 2.7. Assessment of Biological Toxicity of Degraded Products

At the lowest tested concentration of AFB1 (50 µg/L) and AFB2 (20 µg/L), a significant increase in larval mortality was observed compared to the control, i.e., 75%. This percentage of mortality was increased to 87.5% with an increase in incubation time. The mortality response of larvae varied according to the dosage concentration in an experimented period of 24 to 96 h. Larvae treated with dosage concentrations of 100 (AFB1), 50 (AFB2), 200 (AFB1) and 90 µg/L (AFB2) showed 83–91.7% and 86.7–96.7% mortality after 24 to 96 h of incubation, respectively. Similarly, at the highest tested concentration of 300 µg/L AFB1 and 130 µg/L AFB2, the percentage of larval mortality was found to be 95–100% after 24 to 96 h, respectively (Table 5).

**Figure 13:**
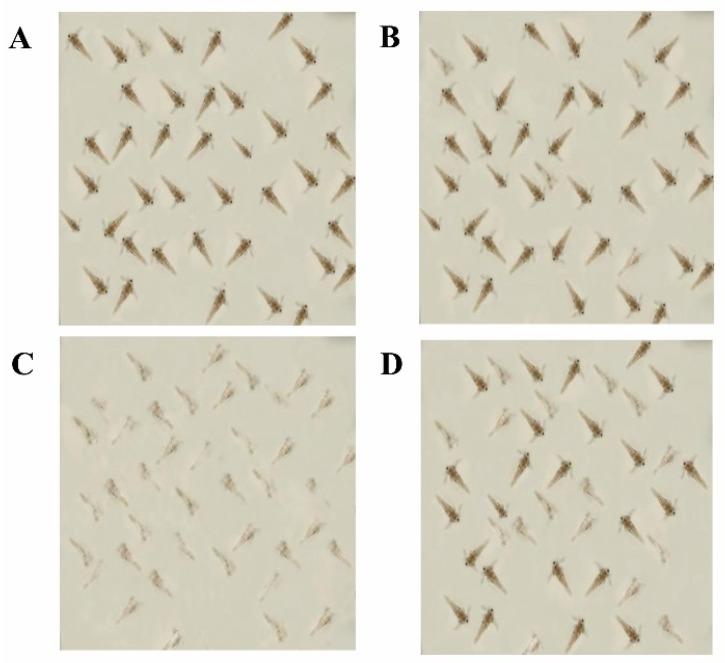
Comparison of toxicity of treated and untreated toxins at concentrations of 300 µg/L (AFB1) and 130 µg/L (AFB2) towards brine shrimp larvae after 96 h of incubation. The effect of different treatments on the mortality of larvae incubated with (**A**): Seawater (control); (**B**): Methanol + seawater; (**C**): Untreated aflatoxins; (**D**): Toxins treated with *M. arvensis* leaf extract.

A significant reduction in larval mortality was recorded after incubation with treated toxins in comparison to the ones incubated with untreated toxins (Figure 13). Larvae incubated with treated 100 µg/L of AFB1 and 50 µg/L of AFB2 showed a percentage mortality of 19.2% after 24 h that increased to 28.3% after 96 h of incubation. This percentage mortality was 63% lower than when shrimps were incubated in a similar concentration of untreated toxins.

## 3. Discussion

Biodegradation of mycotoxins using fungi, bacteria and enzymes is now a known strategy to manage this issue [25,26,27,28,29]. However, not much data is available on the use of plant extracts for the same purpose. In our previous study, aqueous extracts of *Acacia nilotica* were used to detoxify aflatoxins in maize [30]. Whereas, this study was planned to explore the potential of a common medicinal herb, i.e., *Mentha arvensis,* to detoxify or inactivate aflatoxins in maize. In various trials, *M. arvensis* significantly degraded both AFB1 and AFB2 even when checked after three hours of incubation at the lowest tested temperature and pH. However, the percentage of degradation increased with increases in incubation time, temperature and pH.

The high temperature always supports chemical reactions. In in vitro assays, an increase in temperature significantly increased the percentage of degradation of aflatoxins. However, the overall results could be due to the synergistic effect of moisture and high temperature [31]. Many earlier workers have reported similar effects of high temperature on the rate of degradation of mycotoxins [18,32,33]. However, for further in vitro trials, 30 °C was selected to eliminate any bad effect of high temperature on food chemistry. Moreover, 30 °C is quite close to the room temperature and prevailing temperature of storehouses in Pakistan during summer. This selection can make degradation a cost-effective approach at the industrial level.

After temperature, pH also plays an important role in governing a reaction speed. In this study, basic pH supported degradation reactions. Méndez-Albores, et al. [34] showed that a decrease in aflatoxins fluorescence due to a change in coumarin moiety in alkaline conditions. In the present investigation, the highest degradation was achieved at pH 10. However, pH 8 was selected for further trials to avoid the possibility of aflatoxins being unstable and sensitive at the basic pH. As pH 8 is 100 times less alkaline than pH 10 and the percentage of biodegradation was also found comparable to pH 10, pH 8 was preferred for in vitro trials using detached maize stock.

Aflatoxin B1 was converted into seven different compounds. A total of 28% of these compounds were formed due to the modification of the lactone ring. The lactone ring is responsible for the toxicity, carcinogenicity and fluorescence of aflatoxin B1 [35]. Modification in the lactone ring of AFB1 results in the loss of these properties, as observed in this study. Another 28% were formed due to the removal of the methoxy group from the side chain of benzene. A total of 14% of compounds were produced after the removal of the double bond in the furan ring. The presence of the double bond in the furan ring is another feature of AFB1 responsible for its toxicity and carcinogenic properties [36]. The rest of the compounds were formed by the loss of H_2_ and CO_2_.

Aflatoxin B2 was degraded to three different compounds by the replacement of the methoxy group with a hydroxyl group, the addition of H_2_ and removal of CO from the lactone ring and the side chain of the benzene ring. All these changes synergistically reduced the toxicity of the toxin significantly.

These biodegraded products were tested for biological toxicity against brine shrimps. Earlier studies have shown that the eggs and larvae of brine shrimps are susceptible to mycotoxins and hence have been used by several workers as a biological indicator of toxicity of mycotoxins in food and feed [37,38]. The percentage mortality of brine shrimps significantly decreased when they were incubated in water containing treated toxins. Hence it was confirmed that the treatment of selected toxins with aqueous extracts of *M. arvensis* can biodegrade targeted toxins into significantly less toxic compounds. Due to its efficacy, leaves of *M. arvensis* or its aqueous extract can be used as additives in food and feed for making bioactive packaging and mycotoxins binding commercial products to minimize the production and toxicity of aflatoxins. The identification of bioactive components from tested extracts and their use can increase the percentages of biodegradation.

## 4. Materials and Methods

### 4.1. Preparation of Plant Extract

Plants of *Mentha arvensis* were collected from Islamabad, Pakistan, in the month of May. Leaves and shoots of *M. arvensis* were first surface sterilized with 1% sodium hypochlorite for 10 min, followed by several washings with sterile distilled water. A total of 10 g of plant material was homogenized with 10 mL sterilized distilled water to obtain the aqueous extract. This mixture was then filtered using a muslin cloth and centrifuged at 14,000 rpm for 20 min. The supernatant obtained was sterilized by passing through a 0.2 µm syringe filter assembly before using in further trials.

### 4.2. In Vitro Toxin Inactivation Assay

Firstly, the working solution was prepared consisting of methanol and water (60:40, *v*/*v*). A total of 50 μL of the working solution containing 50 μg/L AFB2 and 100 μg/L AFB1 mixed with 250 μL of *M. arvensis* aqueous extract was incubated for various time intervals. After the incubation period, 500 µL of chloroform was added to terminate the reaction. The mixture was thoroughly vortexed to extract the residual toxin. This was followed by the separation of the chloroform fraction at low-speed (5000 rpm) centrifugation. The organic phase was then evaporated to dryness under a gentle stream of nitrogen and dissolved in 100 µL of methanol. A total of 50 μL of toxin in 250 μL of water acted as the control that was also incubated under parallel conditions.

### 4.3. Estimation of Optimal pH for In Vitro Biodegradation Using Plant Extracts

The pH of the aqueous extracts was adjusted between 2.0 and 10.0 using 1N HCl or 1N NaOH and then checked for their biodegradation potential. Distilled water and untreated extract acted as a control.

### 4.4. Estimating Optimal Temperature and Incubation Time for In Vitro Biodegradation Using Plant Extracts

Plant extracts were incubated with toxins at 25, 30, 35, 40, 45, 50, 55 and 60 °C for 3, 6, 12, 24, 48 and 72 h. The toxin content in various reaction mixtures was estimated using high-performance liquid chromatography.

### 4.5. In Vitro Biodegradation of Toxins in Maize Samples Using Plant Extracts

Maize stock samples containing mature grains were spiked with aflatoxins (B1 100 μg/L and B2 50 μg/L). First, samples were decontaminated as described by Das and Mishra [39] with minor modifications. Briefly, 10 g of maize samples were kept in each 250 mL Erlenmeyer flask and spiked with 3 mL of aflatoxins (with concentration B1 100 μg L^−1^ and B2 50 μg L^−1^). These samples were then incubated with 10 mL of aforementioned plant extracts at 30 °C for 72 h. Afterward, aflatoxin was extracted according to the modified method of Stroka, et al. [40]. Maize samples were incubated with water:acetonitrile (15:85%) in a shaking water bath for 2 h. Afterward, the extracts were filtered through filter paper (Whatman, Inc., Clifton, NJ, USA). Afterward, the filtrate was passed through an afla immunoaffinity column in a solid-phase extraction assembly. The toxins were slowly eluted from the column with 1 mL of methanol in a glass vial, which was further analyzed by TLC and HPLC. The control comprised an untreated maize sample, a sample with plant extract without toxin and a sample with toxin without plant extract. Each experiment was performed in triplicate.

### 4.6. Detection and Quantification of Treated Toxins

TLC was used to detect the products of treated toxins. Chloroform and methanol fraction (20 μL), of both treated and control toxins, were run on 0.25 mm silica gel (60F254 20 × 20 cm, Merck, Darmstadt, Germany) TLC plates. These plates were then developed in 92:8 *v*/*v* chloroform:acetone and viewed under UV light (365 nm). Quantification of treated and untreated toxins after derivatization was performed through Agilent 1100 series HPLC (Agilent Technologies, Santa Clara, CA, USA) fitted with a reversed-phase C18 column (Merck, Darmstadt, Germany) and a fluorescence detector. Water:methanol:acetonitrile (60:20:20) was used as the mobile phase with a flow rate of 1 mL/min. Aflatoxins were detected at excitation and emission wavelengths of 360 and 440 nm, respectively. Using a series of calibration solutions in methanol, calibration curves were drawn for HPLC method validation. Each standard solution was chromatographed in duplicate. The identification of degraded toxin metabolites was performed by mass spectral studies.

### 4.7. LCMS Analysis of Bio-Degraded Toxins

Analyses of the degraded toxins and their products were carried out by a surveyor LC system equipped with a mass spectrophotometer and PDA plus detectors (Thermo Fisher Scientific Waltham, MA, USA). All analyses were performed in triplicate using a luna phenomenex C18 column (150 × 4.6 mm, 3 μm), in isocratic mode. The column temperature was maintained at 30 °C. The injection volume was 10 μL, whereas the mobile phase consisted of methanol:acetonitrile:water in 22.5:22.5:55.0 *v*/*v* ratio. The flow rate was 0.5 mL/min. The capillary temperature was 335 °C, sheath gas flow and auxiliary gas flow were 20 and 4 L/min, respectively. Source voltage, capillary voltage and tube lens voltage were 5 KV, 49 V and 120 V, respectively.

### 4.8. ESI–MS/MS Conditions for Aflatoxins through Direct Insertion Pump

Mass spectrometry/mass spectrometry was carried out using a Thermo Scientific LTQ XL System equipped with electrospray ionization (ESI) source operating in the positive ionization mode with a capillary voltage of 49.0 V, a source voltage of 5.0 KV, a tube lens voltage of 110 V and a capillary temperature of 275 °C. The sheath and auxiliary gas flow were adjusted to 3 and 0.4 L/min, respectively, to get a stable spray. Data were collected in positive mode within the range of 100 to 500 *m*/*z*. The identification of unknown compounds was based on the accurate measurement of the mass of parent ions and fragments, as well as other useful MS/MS spectrum information.

### 4.9. Determining Bio-Toxicity of Degraded Products

The bio-toxicity of degraded toxin products was checked using a brine shrimp (Artemia salina) bioassay following the method developed by Solis 36, with some modifications. Brine shrimp eggs, 100–200 mg, were hatched in artificial seawater (34g sea salt/L of deionized water) by incubation at 26 °C under a 60 W lamp. After separating from shells, the nauplii were transferred to fresh seawater. A total of 300 μL of treated and untreated AFB1 (50, 100, 200, 300 μg/L) and AFB2 (20, 50, 90, 130 μg/L) was added to 96-well plates and dried overnight. After solvent evaporation, the toxins were redissolved in seawater (200 μL). Another 200 μL of seawater containing 40–45 organisms was pipetted into each well, making a final volume of 400 μL and were incubated at 26 °C for 24–96 h. Methanol was used as positive control, while for the negative control, seawater was used. Mortality was calculated by counting the immobile (dead) larvae under a stereomicroscope. The test was performed in triplicate.

### 4.10. Statistical Analysis

Five replicates were performed of each treatment, and the values are presented as mean ± standard error. Data acquired in the above experiments were subjected to statistical analyses using DSSTAT software. Analysis of variance (ANOVA) and differences among the means was calculated for significance at *p* ≤ 0.05 using Tukey’s multiple range test.

## Figures and Tables

**Table 1:** Effect of Temperature on Biodegradation by *Mentha arvensis* extracts.

Treatments	Temp (°C)	% Degradation of AFB1						% Degradation of AFB2					
		3 h	6 h	12 h	24 h	48 h	72 h	3 h	6 h	12 h	24 h	48 h	72 h
Toxin	25	0.3 ± 0.0 ^c^	0.8 ± 0.0 ^c^	1.6 ± 0.9 ^b^	1.9 ± 0.1 ^b^	2.2 ± 0.1 ^a^	2.9 ± 0.2 ^a^	0.1 ± 0.0 ^c^	0.5 ± 0.0 ^b,c^	0.7 ± 0.0 ^a–c^	0.8 ± 0.0 ^a–c^	1.0 ± 0.0 ^a,b^	1.4 ± 0.1 ^a^
	30	0.8 ± 0.0 ^d^	0.8 ± 0.0 ^d^	1.8 ± 0.6 ^c^	2.3 ± 0.1 ^b^	3.1 ± 0.2 ^a^	3.8 ± 0.1 ^a^	0.1 ± 0.0 ^c^	0.6 ± 0.0 ^b,c^	0.8 ± 0.0 ^b,c^	0.9 ± 0.1 ^a,b^	1.1 ± 0.0 ^a,b^	1.6 ± 0.1 ^a^
	35	1.2 ± 0.3 ^c^	2.5 ± 0.3 ^b,c^	2.5 ± 0.1 ^b,c^	3.0 ± 0.2 ^b^	3.8 ± 0.2 ^a,b^	4.5 ± 0.3 ^a^	0.2 ± 0.0 ^c^	0.8 ± 0.0 ^b,c^	0.8 ± 0.0 ^b,c^	1.1 ± 0.1 ^b^	1.2 ± 0.1 ^a,b^	1.8 ± 0.2 ^a^
	40	2.2 ± 0.1 ^d^	3.5 ± 0.4 ^c^	3.8 ± 0.2 ^c^	4.3 ± 0.5 ^b^	4.4 ± 0.3 ^b^	5.2 ± 0.4 ^a^	0.3 ± 0.0 ^c^	0.9 ± 0.1 ^b,c^	0.9 ± 0.0 ^b,c^	1.3 ± 0.1 ^b^	1.4 ± 0.1 ^a,b^	2.0 ± 0.1 ^a^
	45	3.2 ± 0.3 ^c^	4.5 ± 0.3 ^b^	5.1 ± 0.3 ^a,b^	5.2 ± 0.4 ^a,b^	5.6 ± 0.5 ^a^	5.8 ± 0.3 ^a^	0.3 ± 0.0 ^c^	1.0 ± 0.2 ^b,c^	1.1 ± 0.1 ^b,c^	1.4 ± 0.1 ^b^	1.6 ± 0.1 ^b^	2.3 ± 0.2 ^a^
	50	4.2 ± 0.5 ^c^	5.5 ± 0.2 ^b^	5.8 ± 0.2 ^b^	6.5 ± 0.3 ^a,b^	6.5 ± 0.4 ^a,b^	6.9 ± 0.4 ^a^	0.3 ± 0.0 ^c^	1.1 ± 0.1 ^b,c^	1.2 ± 0.1 ^b^	1.6 ± 0.1 ^b^	1.7 ± 0.1 ^b^	2.5 ± 0.2 ^a^
	55	5.2 ± 0.3 ^c^	6.4 ± 0.7 ^b^	6.5 ± 0.4 ^b^	7.1 ± 0.5 ^a,b^	7.8 ± 0.6 ^a^	7.9 ± 0.6 ^a^	0.4 ± 0.0 ^d^	1.1 ± 0.1 ^c^	1.4 ± 0.1 ^b,c^	1.7 ± 0.1 ^b^	1.9 ± 0.1 ^b^	2.7 ± 0.1 ^a^
	60	6.2 ± 0.4 ^b^	7.1 ± 0.5 ^a,b^	7.5 ± 0.8 ^a,b^	7.7 ± 0.8 ^a^	7.8 ± 0.4 ^a^	7.9 ± 0.5 ^a^	0.5 ± 0.0 ^d^	1.2 ± 0.2 ^c,d^	1.5 ± 0.1 ^c^	1.8 ± 0.2 ^b,c^	2.0 ± 0.1 ^b^	3.0 ± 0.2 ^a^
Toxin + H_2_O	25	0.2 ± 0.0 ^d^	1.4 ± 0.1 ^c^	2.7 ± 0.2 ^b^	3.4 ± 0.2 ^a^	3.4 ± 0.2 ^a^	3.4 ± 0.2 ^a^	0.3 ± 0.0 ^c^	0.4 ± 0.0 ^c^	1.3 ± 0.0 ^b,c^	1.4 ± 0.1 ^a–c^	2.2 ± 0.1 ^a,b^	2.4 ± 0.1 ^a^
	30	1.1 ± 0.1 ^d^	2.5 ± 0.1 ^c^	3.2 ± 0.3 ^b^	3.5 ± 0.3 ^b^	3.8 ± 0.2 ^a,b^	4.2 ± 0.3 ^a^	0.3 ± 0.0 ^d^	1.1 ± 0.0 ^c^	1.5 ± 0.0 ^c^	2.0 ± 0.1 ^b,c^	2.3 ± 0.2 ^b^	3.4 ± 0.2 ^a^
	35	2.4 ± 0.2 ^d^	3.4 ± 0.2 ^c^	4.7 ± 0.2 ^b^	5.1 ± 0.3 ^a,b^	5.5 ± 0.3 ^a^	5.7 ± 0.3 ^a^	1.2 ± 0.1 ^c^	1.2 ± 0.1 ^c^	2.2 ± 0.1 ^b,c^	2.7 ± 0.2 ^a,b^	2.8 ± 0.1 ^a,b^	3.3 ± 0.2 ^a^
	40	3.7 ± 0.2 ^d^	4.7 ± 0.5 ^c^	6.1 ± 0.5 ^b^	6.4 ± 0.4 ^a,b^	6.8 ± 0.7 ^a^	6.8 ± 0.5 ^a^	2.1 ± 0.3 ^c^	2.7 ± 0.1 ^b^	3.3 ± 0.2 ^a,b^	3.4 ± 0.2 ^a,b^	3.7 ± 0.2 ^a^	3.8 ± 0.3 ^a^
	45	5.1 ± 0.4 ^d^	5.9 ± 0.3 ^c^	7.2 ± 0.6 ^b,c^	7.4 ± 0.5 ^b,c^	7.8 ± 0.5 ^b^	8.0 ± 0.4 ^a^	2.7 ± 0.2 ^d^	3.9 ± 0.2 ^c^	4.1 ± 0.3 ^b,c^	4.1 ± 0.3 ^b,c^	4.5 ± 0.1 ^a,b^	4.9 ± 0.2 ^a^
	50	6.4 ± 0.3 ^d^	7.3 ± 0.6 ^c^	8.3 ± 0.6 ^b^	8.9 ± 0.6 ^b^	9.1 ± 0.7 ^a,b^	9.5 ± 0.8 ^a^	3.4 ± 0.5 ^c^	4.4 ± 0.3 ^c^	4.4 ± 0.1 ^c^	5.6 ± 0.2 ^b^	5.6 ± 0.4 ^b^	6.0 ± 0.4 ^a^
	55	7.7 ± 0.6 ^d^	8.7 ± 0.5 ^c^	9.3 ± 0.8 ^b,c^	9.9 ± 0.7 ^b^	10 ± 1.3 ^a,b^	10 ± 0.6 ^a^	4.2 ± 0.3 ^c^	4.8 ± 0.2 ^c^	4.9 ± 0.3 ^c^	6.7 ± 0.4 ^b^	7.1 ± 0.6 ^a^	7.1 ± 0.3 ^a^
	60	9.1 ± 0.7 ^d^	10 ± 1.1 ^c,d^	10 ± 0.9 ^c,d^	10 ± 0.8 ^c^	11 ± 1.1 ^b^	12 ± 1.1 ^a^	4.9 ± 0.6 ^d^	5.2 ± 0.4 ^c,d^	5.4 ± 0.4 ^c^	7.8 ± 0.7 ^b^	8.3 ± 0.5 ^a,b^	8.6 ± 0.5 ^a^
Toxin + leaf extract	25	44 ± 2.3 ^d^	46 ± 2.3 ^d^	52 ± 3.6 ^c,d^	60 ± 4.3 ^b,c^	66 ± 4.3 ^a,b^	70 ± 6.3 ^a^	62 ± 5.6 ^c^	65 ± 4.3 ^b,c^	66 ± 4.6 ^b^	69 ± 5.7 ^a,b^	70 ± 5.0 ^a^	71 ± 4.3 ^a^
	30	49 ± 4.1 ^d^	52 ± 4.1 ^d^	58 ± 2.3 ^c,d^	61 ± 5.1 ^b,c^	67 ± 5.0 ^a,b^	72 ± 5.9 ^a^	63 ± 7.2 ^d^	65 ± 5.2 ^c,d^	66 ± 5.2 ^c^	70 ± 6.1 ^b^	72 ± 6.2 ^a,b^	74 ± 6.3 ^a^
	35	53 ± 3.2 ^e^	57 ± 3.2 ^d,e^	60 ± 4.1 ^c,d^	64 ± 3.6 ^b,c^	68 ± 3.3 ^a,b^	74 ± 5.0 ^a^	65 ± 4.2 ^d^	66 ± 5.3 ^c,d^	67 ± 4.9 ^c^	72 ± 4.8 ^b^	74 ± 5.8 ^a,b^	75 ± 5.2 ^a^
	40	61 ± 5.1 ^e^	64 ± 5.4 ^d,e^	67 ± 5.2 ^c,d^	71 ± 4.1 ^b,c^	77 ± 6.1 ^a,b^	81 ± 7.1 ^a^	66 ± 4.0 ^d^	67 ± 4.0 ^d^	69 ± 4.8 ^c^	74 ± 6.2 ^b^	76 ± 6.3 ^a,b^	77 ± 4.9 ^a^
	45	62 ± 3.7 ^e^	66 ± 2.9 ^d,e^	69 ± 3.7 ^c,d^	73 ± 6.0 ^b,c^	79 ± 4.9 ^a,b^	85 ± 6.2 ^a^	68 ± 5.9 ^d^	69 ± 5.1 ^d^	71 ± 6.6 ^c^	75 ± 5.3 ^b^	77 ± 4.9 ^a,b^	78 ± 5.8 ^a^
	50	63 ± 4.1 ^e^	66 ± 4.0 ^d^	70 ± 6.1 ^c^	74 ± 5.1 ^b,c^	80 ± 5.3 ^b^	86 ± 7.7 ^a^	69 ± 3.7 ^d^	72 ± 4.9 ^c,d^	73 ± 5.8 ^c^	78 ± 6.1 ^b^	79 ± 5.7 ^a,b^	80 ± 6.2 ^a^
	55	65 ± 5.5 ^e^	68 ± 5.1 ^d^	72 ± 4.5 ^c,d^	76 ± 6.9 ^b,c^	82 ± 4.2 ^a,b^	87 ± 4.9 ^a^	71 ± 4.8 ^d^	72 ± 2.6 ^c,d^	74 ± 4.8 ^b^	79 ± 5.6 ^a,b^	79 ± 5.8 ^a,b^	81 ± 5.4 ^a^
	60	67 ± 4.2 ^d^	68 ± 7.1 ^d^	73 ± 5.1 ^c,d^	78 ± 5.1 ^b,c^	84 ± 6.7 ^a,b^	88 ± 6.2 ^a^	72 ± 5.9 ^d^	73 ± 5.9 ^c,d^	74 ± 5.7 ^c^	80 ± 6.2 ^b^	82 ± 6.3 ^a,b^	83 ± 7.7 ^a^
Toxin + shoot extract	25	19 ± 1.3 ^d^	23 ± 1.9 ^c^	25 ± 2.1 ^b,c^	28 ± 1.3 ^b^	30 ± 2.2 ^a,b^	32 ± 2.6 ^a^	25 ± 1.9 ^f^	29 ± 1.6 ^e^	32 ± 1.9 ^d^	38 ± 2.7 ^c^	43 ± 3.3 ^b^	46 ± 3.6 ^a^
	30	25 ± 2.2 ^d,c^	23 ± 1.6 ^d^	26 ± 1.8 ^c^	29 ± 1.1 ^b,c^	31 ± 1.9 ^b^	34 ± 2.7 ^a^	26 ± 2.3 ^f^	30 ± 2.3 ^e^	33 ± 2.0 ^d^	39 ± 2.8 ^c^	45 ± 2.8 ^b^	48 ± 2.7 ^a^
	35	29 ± 1.7 ^c^	32 ± 2.5 ^b,c^	33 ± 2.3 ^a,b^	35 ± 2.4 ^a^	36 ± 3.4 ^a^	36 ± 3.2 ^a^	28 ± 1.7 ^f^	32 ± 1.9 ^d,e^	35 ± 2.2 ^d^	45 ± 3.3 ^c^	48 ± 3.1 ^b^	50 ± 4.1 ^a^
	40	36 ± 1.6 ^c^	39 ± 2.3 ^b^	41 ± 2.9 ^a,b^	42 ± 3.2 ^a,b^	42 ± 2.8 ^a^	43 ± 3.3 ^a^	29 ± 2.2 ^e^	32 ± 2.6 ^d,e^	34 ± 1.7 ^d^	45 ± 2.1 ^c^	49 ± 3.6 ^b^	51 ± 3.8 ^a^
	45	37 ± 2.8 ^c^	41 ± 3.7 ^b,c^	44 ± 3.6 ^b^	46 ± 3.1 ^a,b^	46 ± 3.1 ^a,b^	47 ± 2.0 ^a^	31 ± 1.9 ^e^	33 ± 1.1 ^d,e^	35 ± 1.8 ^d^	42 ± 1.9 ^c^	48 ± 2.9 ^b^	53 ± 2.7 ^a^
	50	37 ± 2.7 ^c^	41 ± 2.9 ^b,c^	44 ± 2.8 ^b^	46 ± 2.7 ^a,b^	47 ± 2.9 ^a,b^	48 ± 3.9 ^a^	32 ± 2.8 ^e^	33 ± 2.7 ^d,e^	35 ± 2.6 ^d^	43 ± 2.6 ^c^	49 ± 2.7 ^b^	54 ± 3.9 ^a^
	55	40 ± 3.1 ^c^	44 ± 3.1 ^b,c^	45 ± 3.7 ^b^	47 ± 2.9 ^a,b^	48 ± 3.4 ^a,b^	49 ± 2.8 ^a^	34 ± 3.3 ^e^	35 ± 1.9 ^d,e^	37 ± 2.2 ^d^	44 ± 3.1 ^c^	51 ± 4.1 ^b^	56 ± 2.5 ^a^
	60	42 ± 2.8 ^c^	45 ± 2.3 ^b,c^	46 ± 2.6^b^	49 ± 3.2 ^a,b^	50 ± 3.2 ^a^	50 ± 4.0 ^a^	35 ± 1.2 ^e^	35 ± 2.3 ^d,e^	37 ± 2.9 ^d^	45 ± 2.3 ^c^	52 ± 4.4 ^b^	57 ± 3.7 ^a^

Data were analyzed by analysis of variance (ANOVA). Letters in upper case indicate significant differences (*p* < 0.05) among tested plant extracts as calculated by Tukey’s Multiple Range test.

**Table 2:** Effect of pH on biodegradation of AFB1 by leaf and shoot extracts of *Mentha arvensis*.

Treatments	pH	3 h		6 h		12 h		24 h		48 h		72 h	
		Toxin Recovery	D%	Toxin Recovery	D%	Toxin Recovery	D%	Toxin Recovery	D%	Toxin Recovery	D%	Toxin Recovery	D%
Toxin AFB1		99 ± 7.8 ^r^	0.81	99 ± 6.7 ^r^	0.88	98 ± 7.7 ^r^	1.80	97 ± 5.4 ^r^	2.3	96 ± 6.4 ^q,r^	3.07	96 ± 7.5 ^o–r^	3.81
Toxin + H_2_O	pH2	96 ± 9.5 ^p,q,r^	3.2	95 ± 7.3^n –r^	4.6	92 ± 6.9 ^k–p^	7.8	90 ± 6.9 ^g–l^	9.6	89 ± 6.5 ^e–l^	10.2	87 ± 5.9 ^d–j^	12.7
Toxin + H_2_O	pH4	96 ± 7.6 ^o–r^	3.8	95 ± 7.4 ^n–r^	4.7	90 ± 8.8 ^h–m^	9.2	88 ± 5.9 ^d–l^	11.6	87 ± 8.9 ^d–i^	13.0	86 ± 7.9 ^c–h^	13.9
Toxin + H_2_O	pH6	94 ± 5.2 ^m–r^	5.1	92 ± 6.8 ^l–q^	7.5	90 ± 6.8 ^f–l^	9.8	88 ± 7.4 ^c–l^	12.0	86 ± 6.1 ^c–g^	14.0	85 ± 6.0 ^b–f^	14.9
Toxin + H_2_O	pH8	91 ± 6.9 ^j–o^	8.1	89 ± 6.3 ^e–l^	10.1	87 ± 6.1 ^d–k^	12.3	85 ± 5.9 ^b–c^	14.7	84 ± 5.9 ^a–d^	15.9	83 ± 5.7 ^a–d^	16.5
Toxin + H_2_O	pH10	90 ± 6.8 ^i–n^	8.6	87 ± 6.1 ^d–k^	12.2	85 ± 5.0 ^b–c^	14.7	82 ± 5.0 ^a,b,c^	17.7	81 ± 4.5 ^a,b^	18.8	80 ± 5.4 ^a^	19.9
Toxin + H_2_O	WpH	98 ± 5.7 ^r^	1.09	97 ± 5.6 ^r^	2.6	96 ± 5.9 ^p,q,r^	3.23	96 ± 5.8 ^o–r^	3.56	96 ± 5.9 ^o–r^	3.97	95 ± 7.4 ^n–r^	4.19
Leaf extract + AFB1	pH2	56 ± 3.3 ^h–u^	43.8	55 ± 4.4 ^c–u^	44.4	52 ± 2.2 ^a–t^	47.7	48 ± 3.0^a–o^	51.7	42 ± 1.8 ^a–l^	57.5	37 ± 1.6 ^a–j^	62.2
	pH4	54 ± 2.5 ^b–u^	45.7	55 ± 3.5 ^a–u^	44.4	52 ± 3.2 ^a–t^	47.7	48 ± 1.9 ^a–o^	51.7	42 ± 2.9 ^a–j^	57.5	37 ± 2.5 ^a–j^	62.2
	pH6	36 ± 1.6 ^a–q^	64.0	34 ± 2.4 ^a–n^	65.2	30 ± 1.6 ^a–l^	70.0	27 ± 1.9 ^a–l^	72.6	24 ± 0.9 ^a–i^	75.9	20 ± 0.6 ^a–e^	79.6
	pH8	38 ± 3.7 ^a–n^	61.6	34 ± 1.3 ^a–l^	65.7	30 ± 1.5 ^a–k^	69.4	28 ± 1.1 ^a–j^	71.2	23 ± 0.7 ^a–h^	76.8	18 ± 0.4 ^a,b,c^	81.8
	pH10	30 ± 1.5 ^a–j^	69.1	28 ± 1.2 ^a–j^	72.0	26 ± 1.9 ^a–h^	73.9	23 ± 0.8 ^a–g^	76.4	17 ± 1.5 ^a,b^	82.4	12 ± 0.3 ^a^	87.6
	WpH	57 ± 3.4 ^i–u^	42.5	56 ± 3.3^d-u^	43.6	53 ± 3.4 ^b–t^	46.3	50 ± 4.2 ^a–q^	49.8	45 ± 3.1 ^a–m^	54.4	40 ± 1.9 ^a–l^	59.3
Shoot extract + AFB1	pH2	75 ± 4.7 ^q–u^	24.7	76 ± 4.6 ^r–u^	23.7	75 ± 6.5 ^q–u^	24.2	72 ± 5.5 ^k–u^	27.2	66 ± 4.3 ^e–u^	34.0	61 ± 3.6 ^d–u^	38.1
	pH4	77 ± 5.8 ^p–u^	22.6	76 ± 4.7 ^p–u^	23.4	76 ± 4.7 ^o–u^	24.0	69 ± 4.2 ^f–u^	30.8	68 ± 3.5 ^e–u^	31.2	63 ± 5.5 ^d–u^	36.3
	pH6	74 ± 4.3 ^k–u^	25.9	73 ± 6.2 ^j–u^	26.7	71 ± 4.3 ^g–u^	28.5	69 ± 3.2 ^f–u^	30.3	67 ± 3.8 ^e–u^	32.4	62 ± 2.5 ^d–u^	37.1
	pH8	67 ± 3.8 ^e–u^	32.1	66 ± 3.9 ^e–u^	33.6	65 ± 2.6 ^e–u^	34.6	64 ± 4. 1^d–u^	35.8	60 ± 3.5 ^b–u^	39.3	57 ± 3.2 ^a–u^	42.8
	pH10	61 ± 4.7 ^d–u^	38.79	60 ± 4.5 ^d–u^	39.2	58 ± 3.4 ^d–u^	42.3	56 ± 3.2 ^d–u^	43.4	50 ± 4.1 ^a–q^	49.4	43 ± 2.9 ^a–m^	56.9
	WpH	73 ± 4.4 ^j–u^	26.2	71 ± 4.5 ^g–u^	28.8	68 ± 3.8 ^f–u^	31.4	65 ± 5.1 ^e–u^	34.6	62 ± 3.7 ^f–u^	37.3	59 ± 4.3 ^j–u^	40.2

Data were analyzed by analysis of variance (ANOVA). Letters in uppercase indicate significant differences (*p* < 0.05) as calculated by Tukey’s Multiple Range test. D%: Degradation percentage. WpH: without pH adjustment.

**Table 3:** Effect of pH on biodegradation of AFB2 by leaf and shoot extracts of *Mentha arvensis*.

Treatments	pH	3 h		6 h		12 h		24 h		48 h		72 h	
		Toxin Recovery	D%	Toxin Recovery	D%	Toxin Recovery	D%	Toxin Recovery	D%	Toxin Recovery	D%	Toxin Recovery	D%
Toxin AFB1		49 ± 2 ^n^	0.17	49 ± 2 ^l–n^	0.68	49 ± 2 ^k–n^	0.79	49 ± 3 ^h–n^	0.99	49 ± 1 ^g–n^	1.16	49 ± 2 ^d–l^	1.67
Toxin + H_2_O	pH2	49 ± 1 ^n^	1.7	49 ± 2 ^m,n^	1.3	49 ± 7 ^k–n^	4.0	49 ± 3 ^j–n^	4.2	49 ± 3 ^i–n^	5.8	49 ± 3 ^g–n^	5.4
Toxin + H_2_O	pH4	49 ± 3 ^m,n^	2.8	49 ± 3 ^l–n^	3.3	49 ± 3 ^e–n^	6.8	49 ± 4 ^d–n^	7.1	49 ± 2 ^d–m^	7.6	49 ± 2 ^d–l^	9.7
Toxin + H_2_O	pH6	49 ± 3 ^f–n^	6.5	49 ± 2 ^f–n^	6.4	48 ± 3 ^c–k^	10.9	48 ± 2 ^c–i^	10.9	49 ± 3 ^c–h^	10.3	48 ± 3 ^c–h^	11.0
Toxin + H_2_O	pH8	49 ± 2 ^d–m^	7.8	48 ± 3 ^b–g^	11.4	48 ± 2 ^a–g^	13.0	48 ± 3 ^a–e^	12.8	48 ± 1 ^a–e^	11.7	48 ± 3 ^a–d^	12.9
Toxin + H_2_O	pH10	48 ± 4 ^a–f^	12.3	48 ± 3 ^a–c^	15.4	48 ± 4 ^a–c^	15.5	48 ± 3 ^a–c^	15.6	48 ± 2 ^a,b^	17.5	48 ± 3 ^a^	17.2
Toxin + H_2_O	WpH	49 ± 2 ^m,n^	0.36	49 ± 3 ^g–n^	1.14	49 ± 3 ^d–m^	1.56	48 ± 3 ^c–j^	2.04	48 ± 3 ^a–f^	2.41	48 ± 2 ^a,b^	3.41
Leaf extract + AFB1	pH2	21 ± 1 ^e–s^	56.9	20 ± 1 ^d–p^	59.9	18 ± 1 ^c–p^	62.9	17 ± 1 ^b–o^	65.8	15 ± 1 ^b–p^	68.8	16 ± 1 ^b–n^	67.7
	pH4	21 ± 1 ^e–q^	58.0	18 ± 1 ^c–p^	62.6	17 ± 1 ^b–o^	64.7	16 ± 1 ^b–n^	66.5	16 ± 1 ^b–n^	67.3	13 ± 0 ^a–f^	72.5
	pH6	19 ± 2 ^d–p^	60.3	18 ± 1 ^c–p^	62.6	16 ± 1 ^b–n^	66.7	16 ± 1 ^b–n^	67.4	15 ± 0 ^b–p^	69.6	13 ± 1 ^a–e^	74.1
	pH8	17 ± 1 ^b–o^	65.5	16 ± 1 ^b–n^	67.8	13 ±1 ^a–f^	73.0	12 ± 1 ^a–e^	75.2	12 ± 1 ^a–e^	76.0	07 ± 0.6 ^a,b^	84.1
	pH10	15 ± 1 ^b–l^	69.5	13 ± 1 ^a–f^	72.5	10 ± 0 ^a–d^	79.2	9.0 ± 0 ^a–c^	81.9	08 ± 0.3 ^a,b^	83.6	07 ± 0.4 ^a^	85.2
	WpH	18 ± 0 ^c–p^	63.2	19 ± 2 ^d–p^	61.0	17 ± 1 ^b–o^	64.8	16 ± 1 ^b–n^	66.4	15 ± 1 ^b–p^	69.2	13 ± 1 ^a–f^	72.8
Shoot extract + AFB1	pH2	39 ± 2 ^y^	21.7	37 ± 2 ^x,y^	24.9	35 ± 2 ^v–y^	28.4	35 ± 2 ^u–y^	29.9	34 ± 2 ^s–y^	30.9	32 ± 2 ^q–y^	35.1
	pH4	37 ± 3 ^x,y^	24.6	35 ± 1 ^u–y^	29.3	32 ± 2 ^q–y^	35.0	30 ± 3 ^p–y^	38.8	29 ± 1 ^l–x^	42.0	26 ± 1 ^j–x^	46.2
	pH6	36 ± 2 ^v–y^	27.8	33 ± 2 ^r–y^	32.5	30 ± 1 ^p–y^	38.2	29 ± 1 ^l–x^	41.9	27 ± 2 ^j–x^	45.1	25 ± 1 ^h–x^	49.3
	pH8	35 ± 2 ^u–y^	29.2	33 ± 2 ^q–y^	34.0	30 ± 2 ^n–y^	39.7	28 ± 2 ^k–x^	43.4	26 ± 1 ^i–x^	46.6	24 ± 1 ^g–x^	50.8
	pH10	34 ± 1 ^s–y^	31.9	31 ± 1 ^p–y^	36.7	28 ± 1 ^k–x^	42.4	27 ± 1 ^j–x^	46.1	25 ± 1 ^h–x^	49.3	23 ± 2 ^f–x^	53.5
	WpH	36 ± 2 ^w–y^	27.6	34 ± 2 ^s–y^	31.8	33 ± 2 ^q–y^	33.2	32 ± 2 ^q–y^	35.6	30 ± 2 ^p–y^	38.8	28 ± 1 ^k–x^	43.0

Data were analyzed by analysis of variance (ANOVA). Letters in upper case indicate significant differences (*p* < 0.05) among tested plant extracts, as calculated by Tukey’s Multiple Range test. D%: degradation percentage. WpH: without pH.

**Table 4:** In vitro biodegradation of AFB1 and AFB2 at pH 8 and 30 °C after 72 h of incubation.

Treatments	Toxin Recovery (µg/L)	
	AFB1	AFB2
Unspiked maize	0.5 ± 0.02 ^f^	00.3 ± 0.01 ^e^
Unspiked maize + leaf extract	00.0 ± 0.0 ^g^	00.0 ± 0.0 ^f^
Unspiked maize + shoot extract	00.0 ± 0.0 ^g^	00.0 ± 0.0 ^f^
Spiked maize with AFB1 (100 µg/L) and AFB2 (50 µg/L)	97.3 ± 6.7 ^a^	47.6 ± 2.4 ^b^
Spiked maize with toxin + leaf extract	24.9 ± 1.3 ^e^	09.6 ± 0.8 ^d^
Degradation %	75.1 ± 4.9 ^b^	80.8 ± 5.9 ^a^
Spiked maize with toxin + shoot extract	60.0 ± 3.8 ^c^	26.7 ± 1.3 ^c^
Degradation %	40.0 ± 2.5 ^d^	46.5 ± 1.8 ^b^

Data were analyzed by analysis of variance (ANOVA). Letters in upper case indicate significant differences (*p* < 0.05) among tested plant extracts, as calculated by Tukey’s Multiple Range test.

**Table 5:** Percent mortality of brine shrimp larvae at 26 °C after 24 to 96 h at various dose levels of treated and untreated aflatoxins.

Treatments	Toxin Conc. µg/L		No. of Living Cells (hours)				No. of Dead Cells (hours)				% Mortality (hours)			
	AFB1	AFB2	24	48	72	96	24	48	72	96	24	48	72	96
Seawater + shrimps	-	-	40 ± 2.1 ^a^	40 ± 2.5 ^a^	40 ± 2.8 ^a^	39 ± 1.3 ^a^	0.1 ± 0.0 ^f^	0.6 ± 0.0 ^e^	0.5 ± 0.0 ^e^	1.1 ± 0.0 ^e^	0.2 ± 0.0 ^f^	0.1 ± 0.0 ^e^	0.2 ± 0.0 ^e^	2.5 ± 0.1 ^e,f^
Methanol + shrimps	-	-	38 ± 2.3 ^a^	38 ± 1.9 ^a^	37 ± 2.0 ^b^	36 ± 2.8 ^a^	1.6 ± 0.0 ^e^	2.2 ± 0.1 ^d^	3.1 ± 0.2 ^d^	3.5 ± 0.1 ^d^	2.5 ± 0.1 ^e^	5.3 ± 0.3 ^d^	7.5 ± 0.4 ^d^	7.5 ± 0.6 ^e^
Untreated toxins + shrimps	50	20	10 ± 0.8 ^b^	07 ± 0.4 ^b^	5.1 ± 4.1 ^c^	5.2 ± 2.6 ^b^	30 ± 1.6 ^c,d^	33 ± 2.2 ^b,c^	35 ± 3.1 ^b,c^	35 ± 2.1 ^b,c^	75 ± 4.2 ^c,d^	81 ± 5.2 ^b,c^	86 ± 6.2 ^b,c^	87 ± 3.9 ^c,d^
	100	50	7.3 ± 0.6 ^b,c^	5.4 ± 0.3 ^c^	4.5 ± 3.4 ^c^	3.7 ± 1.5 ^b,c^	33 ± 1.1 ^b,c^	35 ± 3.1 ^a,b^	36 ± 1.8 ^a,b^	37 ± 1.1 ^a,b^	83 ± 3.8 ^b,c^	86 ± 7.3 ^a,b^	89 ± 5.0 ^a,b^	91 ± 5.2 ^b,c^
	200	90	5.9 ± 0.2 ^c,d^	3.2 ± 0.1 ^c^	2.9 ± 1.2 ^d^	1.9 ± 1.0 ^c,d^	35 ± 2.8 ^a,b^	37 ± 1.6 ^a^	38 ± 1.7 ^a^	39 ± 2.5 ^a^	86 ± 5.9 ^a,b^	91 ± 5.4 ^a^	94 ± 6.3 ^a^	96 ± 5.7 ^b^
	300	130	2.3 ± 0.1 ^e^	2.8 ± 0.1 ^c,d^	1.7 ± 0.8 ^e^	0.3 ± 0.0 ^e^	38 ± 1.7 ^a^	38 ± 2.5 ^a^	39 ± 2.9 ^a^	40 ± 3.1 ^a^	95 ± 7.1 ^a^	95 ± 3.6 ^a^	96 ± 5.8 ^a^	100 ± 7 ^a^
Toxin degraded with *M. arvensis* Leaf extracts + shrimps	50	20	35 ± 2.4 ^a^	33 ± 2.6 ^a^	32 ± 2. 1^a^	31 ± 1.7 ^a^	05 ± 0.3 ^b,c^	07 ± 0.3 ^b,c^	08 ± 0.6 ^b,c^	09 ± 0.5 ^b,c^	13 ± 1.4 ^d^	17 ± 1.3 ^d^	19 ± 1.4 ^d^	22 ± 1.5 ^c,d^
	100	50	32 ± 1.9 ^a^	31 ± 1.7 ^a^	31 ± 1.1 ^a^	29 ± 1.4 ^a^	08 ± 0.7 ^b^	09 ± 0.5 ^b^	09 ± 0.1 ^b^	11 ± 0.7 ^b^	19 ± 1.0 ^c^	21 ± 1.4 ^c^	23 ± 1.9 ^c^	28 ± 1.4 ^c^
	200	90	28 ± 1.3 ^a,b^	27 ± 1.3 ^b^	26 ± 1.3 ^b^	26 ± 0.9 ^a,b^	12 ± 0.9 ^a^	13 ± 0.9 ^a^	14 ± 1.2 ^a^	14 ± 1.1,^a^	30 ± 2.6 ^a,b^	32 ± 2.2 ^a,b^	34 ± 2.3 ^b^	35 ± 2.4 ^b^
	300	130	26 ± 1.9 ^b,c^	25 ± 2.2 ^c^	25 ± 0.9 ^b^	24 ± 1.5 ^b,c^	14 ± 1.1 ^a^	15 ± 1.1 ^a^	15 ± 1.1 ^a^	16 ± 1.3 ^a^	34 ± 2.7 ^a^	36 ± 2.8 ^a^	38 ± 2.6 ^a^	40 ± 2.7 ^a^

Data were analyzed by analysis of variance (ANOVA). Letters in upper case indicate significant differences (*p* < 0.05) among tested plant extracts, as calculated by Tukey’s Multiple Range test.

## Data Availability

Data is contained within the article.
